# High-frequency oscillatory ventilation for PARDS: awaiting PROSPect

**DOI:** 10.1186/s13054-020-2829-3

**Published:** 2020-03-27

**Authors:** Martin C. J. Kneyber, Ira M. Cheifetz, Martha A. Q. Curley

**Affiliations:** 1 Department of Paediatrics, Division of Paediatric Critical Care Medicine, Beatrix Children’s Hospital, University Medical Center Groningen, University of Groningen, Groningen, the Netherlands; 2 Critical Care, Anaesthesiology, Perioperative & Emergency Medicine (CAPE), University of Groningen, Groningen, the Netherlands; 3 Pediatric Acute Lung Injury and Sepsis Investigators, Durham, NC USA; 4 Family and Community Health, School of Nursing, Anesthesia and Critical Care Medicine, Perelman School of Medicine, University of Pennsylvania, Philadelphia, PA USA; 5 Research Institute, Children’s Hospital of Philadelphia, Philadelphia, PA USA

Recently, Wong et al. reported increased 28-day mortality among 328 children with pediatric acute respiratory distress syndrome (PARDS) managed with high-frequency oscillatory ventilation (HFOV) [1]. This study is an excellent example of gaining a better understanding of pediatric critical care through multicenter collaboration. Nonetheless, there are some nuances one should consider before interpreting the study results. Inherent to the study design, confounding by indication (i.e., the sickest patients are those most likely to receive a specific intervention) occurred albeit that the authors used advanced statistical techniques to address this. Furthermore, HFOV was largely employed as rescue without consistent criteria for its use and clinical management was done without a consistent protocol.

Although HFOV has been available for several decades, we have no data demonstrating an optimal physiologic approach to HFOV management in the acute and weaning phase. Most pediatric HFOV papers make no mention of recruitment maneuvers (RMs) and reported frequencies (F) in the range of 5–8 Hz [2]. Yet, optimizing lung volume by means of a RM may be physiologically necessary to recruit collapsed, atelectatic lung units to improve oxygenation and prevent exposure to larger, potentially more injurious pressure swings [3]. Low F is not in line with the concept of the corner frequency (Fc) [4]. Fc is the F with the lowest pressure cost of ventilation and thus the least injurious to the lung. In disease conditions with reduced respiratory system compliance, such as PARDS, Fc is increased indicating that the highest oscillatory F that still allows for adequate ventilation might be preferable. Also, there are no data guiding the HFOV weaning process, possibly explaining the observed increased ventilation times seen in patients managed with the oscillator [5].

We are now enrolling pediatric patients in the Prone and Oscillation Pediatric Clinical Trial (PROSpect) to address the issue surrounding the uncertainty regarding the role and optimal management of HFOV for PARDS. In this adaptive randomized control trial, patients with high moderate-to-severe PARDS (OI > 12) are randomized to test the hypothesis that prone versus supine positioning and HFOV versus conventional mechanical ventilation (CMV) will result in a 2-day improvement in ventilator-free days. In this trial, CMV and HFOV are strictly protocolized, and the HFOV protocol makes use of staircase RMs, high F, and daily titration to improve the weaning process.

From our perspective, until we have the results of this RCT, there is therefore no need to abandon HFOV.

## Data Availability

No applicable
